# At-home dried blood spot (DBS) collection to increase population heterogeneity representation in pediatric research: An ECHO study

**DOI:** 10.3389/fped.2023.1059107

**Published:** 2023-03-03

**Authors:** Joseph M. Green, Fatoumata Barry, Phoebe Burton, Jennifer Beauchemin, Matthew J. Huentelman, Sean C. L. Deoni, Candace R. Lewis, on behalf of program collaborators for Environmental Influences on Child Health Outcomes

**Affiliations:** ^1^ School of Life Sciences, Arizona State University, Tempe, AZ, United States; ^2^ Rhode Island Hospital, Warren Alpert Medical School, Brown University, Providence, RI, United States; ^3^ Neurogenomics, Translational Genomics Research Institute, Phoenix, AZ, United States; ^4^ Department of Pediatrics, Warren Alpert Medical School, Brown University, Providence, RI, United States

**Keywords:** dried blood spot (DBS), population representation, biological samples, at-home DBS collection, remote science

## Abstract

Self-collection of dried blood samples (DBS) in the participant's home provides an alternative to university/hospital visits for research and has the potential to improve the representation of population heterogeneity in research. This study aimed to assess the feasibility of guardian and/or self-DBS collection in healthy youth in the lab and home. Guardians/youth [*N* = 140; females = 63; *M*_age_ = 8.73, *SD*_age_ = 3.56] who enrolled in a longitudinal study of typical development were asked during a lab visit to provide a DBS. Upon providing a sample, the participants were asked if they would be willing to self-collect in the home and return the sample *via* the post office. Of those asked to provide a sample in the lab, 82% consented and 18% declined, with a significant difference in age but no significant difference in sex, ethnicity, race, or family income. Of those who provided a sample in the lab, 75% were willing to self-collect DBS in the home, with no significant difference in demographic variables between them. We report a quality assessment and DNA extraction results from a subset of samples. The results demonstrate a high feasibility of DBS collection from healthy youth for research purposes both in the laboratory and in the home across different demographic variables. Developmental researchers should consider including this approach in their studies to increase population heterogeneity representation.

## Introduction

Dried blood spots (DBS), collected by pricking the finger or heel and dabbing drops of blood on filter paper, can be used as a viable alternative to more expensive and invasive methods of obtaining biological samples. DBS collection is becoming more common and is beneficial in various fields. Specifically, DBS can be used to identify genes and RNA-based biomarkers associated with various neurological and developmental disorders, therapeutic drug monitoring, and protein measurement (1).

An advantage of DBS collection is its practicality in non-clinical settings because a phlebotomist and laboratory equipment are not required. The efficacy of at-home DBS collection has been demonstrated in several clinical pediatric studies, such as those on children with epilepsy (2), autism spectrum disorder and severe behavioral problems (3), children with chronic myeloid leukemia (4), and pediatric kidney transplant recipients (5). However, no studies to date have validated DBS collection from a healthy pediatric population. Clearly, at-home DBS collection is beneficial to researchers and patients alike because of the ease of process and decreased cost; however, there are potentially more advantages in utilizing at-home biological sample collection procedures.

Adaptation to off-site sample collection may provide an unprecedented opportunity to diversify study samples, addressing long-standing calls for increased representation of population heterogeneity in research (6). An expansion of remote data collection was recently catalyzed by the COVID-19 pandemic and demonstrated potential widespread application. Traditional methods of biological sample collection are burdensome to participants as they require time and effort to travel to hospitals/research centers. At-home collection is advantageous because of the possibility of including those not within close proximity to research settings and those with stringent resource limitations. Therefore, this study aimed to assess the (1) feasibility of guardian/self-collection of DBSs in a healthy pediatric population and (2) if demographic variables such as age, sex, ethnicity, race, and family income levels influenced the willingness to provide in-lab or at-home DBS for research purposes.

## Methods

### Parent study

Our study was based on a subset of participants prospectively followed as part of the Environmental influences on Child Health Outcomes (ECHO) Program. ECHO is a consortium of 69 established pediatric cohort studies collecting new data under a common protocol since 2019 (7) with the primary aim of studying the effects of early-life environmental exposures on child health. Single and cohort-specific institutional review boards monitor human subject activities and those of the centralized ECHO Data Analysis Center. All participants provided written informed consent.

The following were the eligibility criteria for the parent study: mothers >18 years old, term gestation 37–41weeks, healthy singleton pregnancy, no evidence of uncontrolled medical conditions (i.e., hypertension, pre-eclampsia, uncontrolled diabetes) or medical conditions that could potentially impact the safety of a participant during a study visit, no history of major psychiatric illness, English–speaking participants, providing consent to baby brain imaging and the longitudinal nature of the study, infants with no signiﬁcant congenital anomalies, and infants with no history of neurological trauma or disorder (e.g., epilepsy). The inclusion criteria for the subset used in this study included participants who came to the lab during the study recruitment period. Participants' recruitment occurred in person at a research visit or remotely.

### Demographics

This study sample included guardians/youth (*N* = 140; females = 63; age range <1–17 years, *M*_age_ = 8.73, *SD*_age_ = 3.56) previously enrolled in a longitudinal ECHO study. The race variable was based on self-report and coded as (code: % of the sample) White (1: 72%); Black or African American (2: 3.5%); Asian (3: 2%); American Indian or Alaska Native (4: 0%); Native Hawaiian or Other Pacific Islander (5: 0%); Multiple Categories Reported (6: 13.5%); Other (7: 3%). The family income variable was based on self-report and coded as (% of the sample) $0–29,999 (1: 8.5%); $30,000–49,999 (2: 13.5%); $50,000–99,999 (3: 19%); $100,000–more (4: 42%).

### Procedures

Upon the completion of study consenting during a lab visit, the participants were asked to provide a DBS in the lab. The lab staff used a BD Microtainer Contact–Activated lancet with a 2.0 mm blade to collect blood for all age groups. Alcohol swabs were used to disinfect the area. For all age groups, the collection site was the pulp of the ring or middle finger of the non-dominant hand. Guardians/youth were allowed to use any preferable means necessary to decrease discomfort, including but not limited to parents holding the child, watching a video or game on a phone/tablet, conversation, etc. If necessary, the collection site was gently “milked” to complete the DBS collection card. Upon providing a DBS sample, the participants completed a survey pertaining to their willingness to self-collect in the home and return the sample *via* the post office. The participants were provided a $50.00 gift card of a popular nationwide store.

### DBS quality assessment and DNA extraction

A subset of DBSs were assessed for sample quality (*n* = 44). Sample quality was determined by the following criteria: (1) Excellent—at least five circles saturated at 80%; Good—at least four circles saturated at 80%; Acceptable—at least four circles saturated at 50%; Poor—at least three circles saturated at 50%. DNA was extracted from this subset of samples following the Automated DNA Purification from Blood on FTA Cards protocol using the Maxwell® RSC FFPE Plus DNA Kit and the Maxwell® RSC Instrument. DNA was quantified using a standard PicoGreen® protocol.

### Statistics

A Student's independent sample t-test was used to compare groups for age. Chi square analysis was used to assess if sex differed between groups. Logistic regression was used to assess if race or ethnicity differed between groups. Logistic regression was used to determine if DBS quality predicted DNA quantity. Participants were excluded from the analysis if any variable was found missing.

## Results

All descriptive statistics for age, sex, ethnicity, race, and family income for the four groups are reported in Table 1.

**Table 1:** Sample demographics by group.

	Provided DBS M (SD)/%	*n*	Declined DBS M (SD)/%	*n*	Willing to self-collect at home	*n*	NOT willing to collect at home	*n*
**Sample**								
Age (years)	9.5 (3.16)	115	5.2 (2.99)	25	9.3 (3.2)	87	9.76 (3.1)	18
Sex (female)	45%	115	44%	25	46%	87	44%	18
**Self-reported ethnicity**								
Hispanic	16%	18	29%	7	16%	14	11%	2
Non-Hispanic	84%	95	71%	17	84%	71	89%	16
**Self-reported race**								
White	74%	85	68%	17	75%	65	66%	12
African American or Black	4.3%	5	0	0	2%	2	6%	1
Asian	2.6%	3	0	0	1%	1	11%	2
American Indian/Alaska Native	0	0	0	0	0	0	0	0
Native Hawaiian or other Pacific Islander	0	0	0	0	0	0	0	0
Multiple categories reported	12%	14	20%	5	16%	14	11%	2
Other	3.4%	4	0%	0	5%	4	0	0
Missing	3.4%	4	12%	3	1%	1	6%	1
**Family income**								
$0–29,999	8%	9	12%	3	8%	7	0	0
$30,000–49,999	14%	16	12%	3	13%	11	22%	4
$50,000–99,999	21%	24	12%	3	25%	22	0	0
$100,000—more	43%	50	36%	9	40%	35	56%	10
Missing	14%	16	28%	7	14%	12	22%	4

### Comparisons between participants who provided a DBS and those who declined

**Figure 1:**
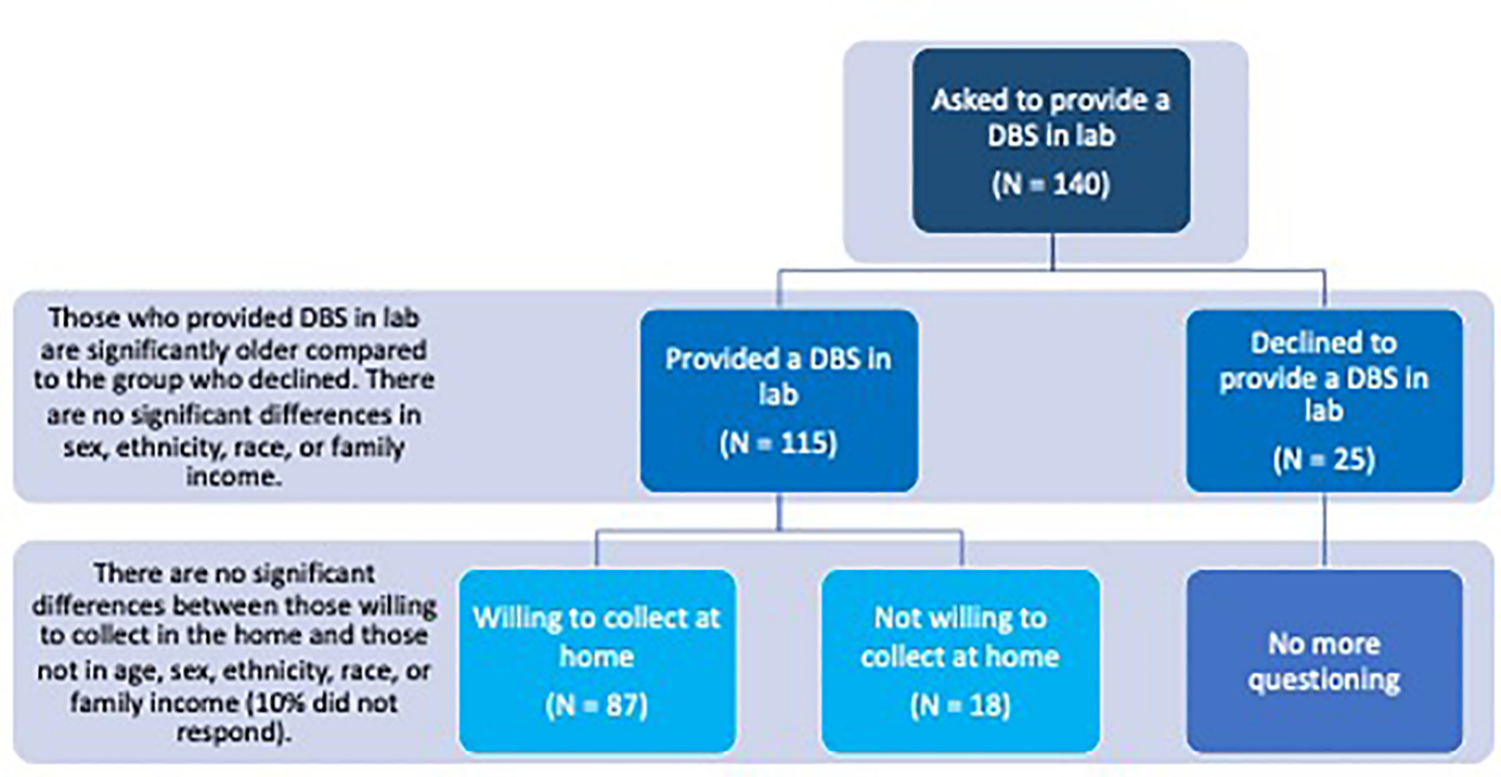
Schematic layout of the study flow and results. Results suggest high feasibility for in-lab and at-home DBS collection in healthy children. Of those asked to provide a sample in the lab (*N* = 140), 82% consented and provided a DBS and 18% declined. Those who provided a DBS sample in lab were significantly older than those who declined. Of those who provided a DBS in the lab (*N* = 115), 75% were willing to self-collect DBS in the home, 17% declined, and 10% did not respond. There was no significant difference in age, ethnicity, race, or family income between those willing to self-collect in the home and those who were not.

**Figure 2:**
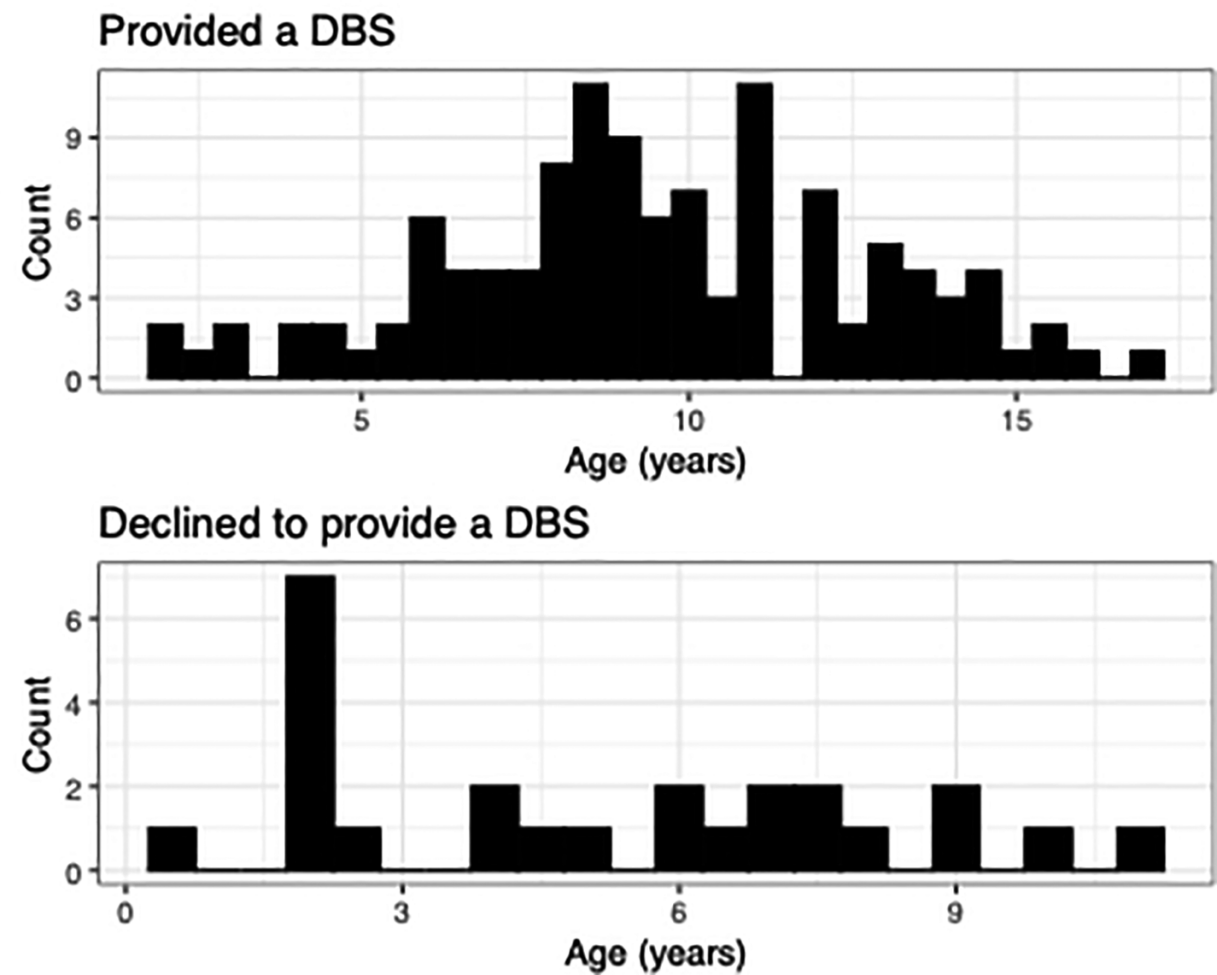
Comparison of ages between families who provided a DBS and those who declined. Age distribution plots highlight the difference in ages between those who provided a DBS and those who refused. Families with younger children were more likely to decline providing a sample.

Of those asked to provide a sample in the lab (*N* = 140), 82% consented [*n* = 115, *M*_age_=9.51, *SD*_age_ = 3.19; female = 52] and 18% declined [*M*_age_ = 5.16, *SD*_age_ = 3.56; female = 11], with a significant difference in age [*t*(138) = −6.24, *p* = 5.024 × 10^−09^] but no significant difference in sex [*X*^2^ (1) = 0.0, *p* = 1], ethnicity (Hispanic or Non-Hispanic) [*β* = 0.13, *p* = 0.13], race [all *p*'s > 0.05], or family income [all *p*'s > 0.05] (Figure 1). Age distributions for those who provided a DBS and those who declined can be found in Figure 2.

### Comparisons between participants willing to self-collect DBS at home and those who declined

Of those who provided a sample in the lab (*N* = 115), 75% were willing to self-collect DBS in the home [*n* = 87, *M*_age_ = 8.69, *SD*_age_ = 3.19; female = 63] [17% declined [*n* = 18, *M*_age_ = 8.73, *SD*_age_ = 3.23; female = 8], 10% did not respond]. There was no significant difference in age [*t*(103) = 0.49, *p* = 0.62), sex [*X*^2^ (1) = 1, *p* = 0.1], ethnicity [*p* = 0.68], race [all *p*'s > 0.05], or family income [all *p*'s > 0.05] between those willing to self-collect in the home and those who were not (Figure 1).

### DBS quality and DNA extraction

DBS quality results can be found in Table 2. The quality of DBS did not predict the amount of DNA extracted [all *p*s > 0.05].

**Table 2:** DBS quality and DNA quantification.

Quality	*n*	%	Mean concentration (ng/μl) (SD)	Mean total DNA (ng) (SD)
Excellent	21	48	17.8 (1.0)	890 (501)
Good	13	30	14.7 (1.0)	732 (343)
Acceptable	5	11	12.5 (1.4)	624 (277)
Poor	5	11	10.1 (0.5)	505 (250)

## Discussion

These results demonstrate a high feasibility of DBS collection from healthy youth for research purposes both in the laboratory and in the home. Our results suggest that especially older children and their guardians are willing to collect DBS at home and return them by mail. We found no difference in terms of the willingness to collect DBS from healthy children at home across different ethnic, racial, or income levels. A visual assessment revealed that most participants provided a high-quality DBS sample. Further, we demonstrated the ability to extract enough DNA for most downstream applications such as microarrays and sequencing.

The limitations of this study include the sampling of a predominantly White population with a high socioeconomic status. Future research with a more diverse sample is needed for producing more generalized findings. We recruited people from a sample that was already involved in research, thereby lending the sample to bias . For this study, we were unable to request at-home DBS collection and used self-report data instead. A follow-up study including actual at-home DBS collection and retention rates would strengthen the conclusions of this preliminary assessment.

At-home DBS collection is an alternative to university/hospital visits for research and has the potential to improve population heterogeneity representation in research. Participants in rural and resource-limited areas or those rendered with difficulties related to travel can participate remotely and send samples by mail to research institutes. Developmental researchers should consider how to include this approach in their studies to increase population heterogeneity representation.

## Data Availability

The raw data supporting the conclusions of this article will be made available by the authors without undue reservation.
